# Recurrent spontaneous pneumothorax and surgical management in Birt–Hogg–Dubé syndrome: a case report

**DOI:** 10.1097/RC9.0000000000000269

**Published:** 2026-02-16

**Authors:** Thomas Roberts, Thomas Fraccalini, Kunal Bhakhri, Alessandro Maraschi

**Affiliations:** a Division of Medical Education, University College London Medical School, London, UK; b Department of Emergency Medicine, San Luigi Gonzaga University Hospital, Orbassano, Turin, Italy; c Thoracic Surgery Department, University College London Hospitals, London, UK

**Keywords:** Birt-Hogg-Dubé syndrome, case report, genetic etiology, spontaneous pneumothorax, talc pleurodesis, VATS bullectomy

## Abstract

**Introduction:**

Birt–Hogg–Dubé (BHD) syndrome is a rare autosomal-dominant disorder caused by a *FLCN* gene mutation, predisposing individuals to renal cancer, pulmonary cysts, and spontaneous pneumothorax. This report highlights the surgical management of recurrent pneumothorax in a patient with BHD.

**Presentation of case:**

A male in his 30s with a recent BHD diagnosis presented with sharp left-sided chest pain. He had a history of pneumothorax since age 14. Imaging confirmed a moderate left-sided pneumothorax with underlying lung cysts. After chest drain insertion, he underwent a successful video-assisted thoracoscopic (VATS) bullectomy and talc pleurodesis. His recovery was uncomplicated, with discharge on day 3.

**Discussion:**

BHD syndrome significantly increases the lifetime risk of spontaneous pneumothorax due to the rupture of characteristic pulmonary cysts. Management is primarily surgical to prevent recurrence, with VATS bullectomy and pleurodesis being the preferred approach due to its efficacy and minimal invasiveness. This case illustrates the typical early onset and recurrent nature of pneumothoraces in patients with a positive family history.

**Conclusion:**

This case underscores the critical importance of considering BHD syndrome in the differential diagnosis for patients, especially younger individuals, with spontaneous or recurrent pneumothorax. Early recognition facilitates appropriate surgical intervention and enables essential screening for associated renal cell carcinoma.

## Introduction

Birt–Hogg–Dubé (BHD) syndrome is a rare autosomal-dominant genodermatosis caused by a mutation in the folliculin (*FLCN*) gene on chromosome 17p11.2^[1,2]^. This tumor suppressor gene mutation predisposes individuals to fibrofolliculomas, renal cell carcinoma, pulmonary cysts, and spontaneous pneumothorax[1]. In the context of BHD syndrome, spontaneous pneumothorax occurs due to the rupture of these characteristic lung cysts[3]. We report a case of recurrent spontaneous pneumothorax managed surgically in a patient with BHD syndrome, in line with the 2025 SCARE criteria[4]. AI was not used in the writing this report. This case was managed at a university teaching hospital and tertiary thoracic surgery referral center.

**HIGHLIGHTS**

- Birt–Hogg–Dubé syndrome is a key genetic cause of spontaneous pneumothorax.
- Surgical management via video-assisted thoracoscopic bullectomy and pleurodesis is the definitive treatment.
- Early recognition allows for crucial renal cancer screening in patients and families.
- Pneumothorax in BHD can occur at a young age, especially with a family history.
- Histology typically shows subpleural blebs and bullae with chronic inflammation.

## Presentation of case

Informed consent was obtained. A male in his 30s (ex-smoker) presented to the emergency department with sharp, non-radiating, left-sided chest pain and decreased breath sounds. He had a recent diagnosis of BHD and a history of a left-sided spontaneous pneumothorax (the first incidence at age 14), previously treated with talc pleurodesis and bullectomy.

**Figure 1.**
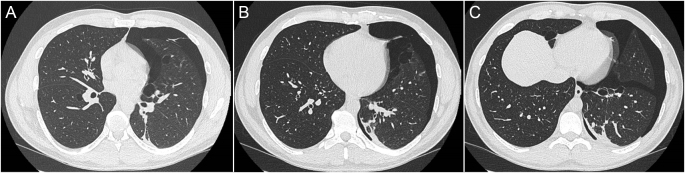
Preoperative CT imaging demonstrating (A) left upper lobe bullae; (B) left upper and right lower lobe bullae; (C) left lower lobe bullae.

A chest X-ray confirmed a moderate left-sided pneumothorax. Subsequent CT imaging revealed a pneumothorax with a maximal apical depth of 33 mm, shallow pleural effusion, and multiple ipsilateral lung cysts and blebs (Fig. 1). A chest drain was inserted. He received prophylactic enoxaparin and analgesia.

**Figure 2.**
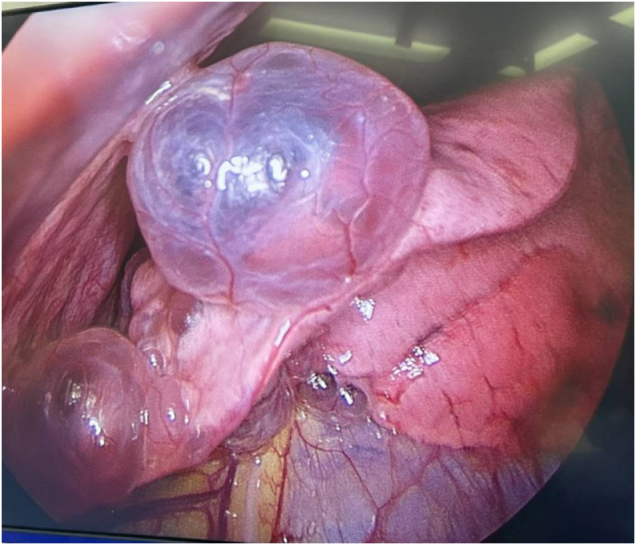
Lingular bulla in the left upper lobe.

Five days following his diagnosis of left-sided moderate-volume pneumothorax with bilateral lung cysts/ blebs, he underwent a video-assisted thoracoscopic (VATS) bullectomy and talc pleurodesis. Adhesions of fibrous tissue were present between the lingula of the left upper lobe, the heart and mediastinum. The adhesions were broken down, and the upper lobe was released to identify left upper lobe bulla at the lingula (Fig. 2). The bullectomy was performed, and the specimen was sent for histopathology on formalin. Eight grams of talc was pulverised throughout the chest cavity, with good talc distribution with good hemostasis observed. A chest drain was inserted, and the lung re-expanding well before chest closure. The procedure went as planned with no changes.

The histology sample taken comprised 61 × 70 x 25 mm lung tissue with an intact bulla on the surface measuring 31 × 25 mm. Microscopically, sections of Lung parenchyma with a subpleural bulla and subpleural blebs showed histiocytic inflammation. Away from the bulla, signs of chronic inflammation were noted on the lung tissue – no evidence of malignancy or granuloma formation was noted. Macroscopic and microscopic findings led to the conclusion of bullous disease from the histopathology report.

**Figure 3.**
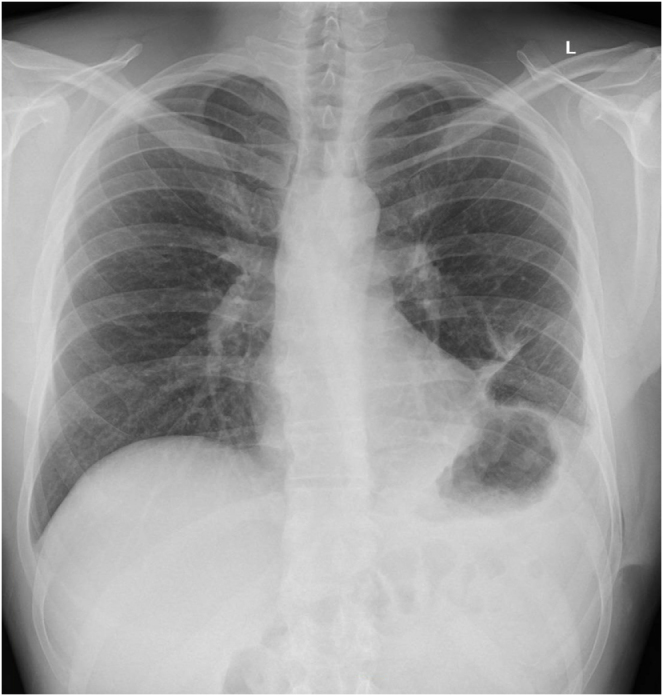
Postoperative chest radiograph following chest drain removal.

Postoperative recovery was uncomplicated, The chest drain was removed 3 days after the surgery, with a satisfactory chest X-ray taken postremoval (Fig. 3). As BHD can also manifest in the form of increased risk of developing benign and malignant kidney tumors, blood tests were taken (sodium, potassium, creatinine, and liver function tests) to examine renal, liver, and bone profile postoperatively. His blood markers were all satisfactory (and as such, further renal imaging was not indicated within this limited follow-up period), and the pain was well controlled with simple analgesia. He was discharged 3 days post-surgery. A follow-up appointment was provided at an outpatient clinic 2 weeks post-discharge.

## Discussion

BHD syndrome, caused by mutations in the *FLCN* gene, disrupts cellular pathways like mTORC1 and AMPK, leading to uncontrolled cell growth[5]. This confers a significantly increased risk of renal cancer (7-fold) and pulmonary cysts (>80% of patients), with a lifetime spontaneous pneumothorax risk of 30%[6].

While benign skin lesions (fibrofolliculomas) are common, renal tumors and pulmonary complications are the primary clinical concerns. Renal tumors, often multifocal and bilateral, develop in 25–35% of patients by age 50^[6,7]^, necessitating regular screening with CT/ MRI.

Pulmonary cysts are more commonly observed than renal tumors in BHD patients, with CT imaging estimating their presence in approximately 85%–87% of cases. BHD-associated pulmonary cysts are a risk factor for spontaneous pneumothorax due to their hamartoma-like composition and high likelihood of rupture, leading to a breach in lung tissue and subsequent air escape into the pleural cavity. These cysts typically appear bilaterally and are located in the lower lobes of the lungs – interestingly, our case presented with both upper and lower lobe pulmonary cysts, with bullae concentrated in the left upper lobe. Incidences of spontaneous pneumothorax in patients with BHD are common, with a 75% chance of experiencing a first pneumothorax by the age of 50; however, the likelihood of a first pneumothorax before age 30 is much lower at around 6% [95% confidence interval (CI), 3%–10%], highlighting the rarity of our case in which both initial and recurrent pneumothorax occurred within this age range. Studies suggest that mutation of the FCLN gene and a family history of spontaneous pneumothorax (as observed in the case described here) significantly increase the risk of developing a spontaneous pneumothorax compared to BHD patients without a family history, possibly explaining the early onset in this case.

Pleuropulmonary lesions associated with BHD have several distinctive histological findings, many of which are described in our case – these include thin-walled pleural and subpleural cysts and bullae, pleural blebs and changes consistent with spontaneous pneumothorax, and underlying emphysematous changes in lung tissue parenchyma adjacent to the bullae[7].

Management of pneumothorax in BHD is surgical to prevent recurrence. Initial chest drainage is followed by VATS, the preferred approach for bullectomy and pleurodesis due to its efficacy and minimal invasiveness[3], as demonstrated in this case. There is no cure for the underlying genetic defect; management focuses on treating its sequelae. The primary limitations of this report are its nature as a single case and the short-term follow-up period, which preclude broader generalizations about long-term outcomes.This case underscores the importance of recognising BHD in patients with spontaneous pneumothorax, especially when recurrent or with a family history, to enable appropriate screening and surgical management.

## Data Availability

The data supporting the findings of this case report are derived from the patient’s clinical records and are not publicly available due to privacy and ethical restrictions. The patient provided informed consent for their de-identified clinical information and images to be used for publication in this research.

## Research registration unique identification number (UIN)

As a case report, this study does not require prospective registration in a public trials registry. Therefore, a unique identification number (UIN) is not applicable.
